# An opportunity and a beginning!

**DOI:** 10.4103/0019-5545.31510

**Published:** 2007

**Authors:** T. S. Sathyanarayana Rao

**Affiliations:** Department of Psychiatry, J. S. S. Medical College and Hospital, Ramanuja Road, Mysore - 570 004, Karnataka, India

“Change is the law of life. Those, who look only to the past or present, are certain to miss the future”.

John F. Kennedy

These thoughts are but loud thinking of a sort. Personally, my being in the field of psychiatry for the last 28 years I have seen the journal grow in leaps and bounds. I bow with reverence to the past editors who have strived to keep the journal in the forefront and as the true indicators of sweeping changes happening in our Indian society in general and the psychiatry, in particular. Quarter century may be a short period in the millennium but too important a time to be missed out. Last few years, in fact the journal has changed quite a bit for good - It can definitely rate among the best publications the world over - talk of the get up, available intellectual input and stimulating content. Hailing from the silicon valley of India, yet I felt we can make use of best available changes around us, particularly the information technology and its varied avatars. That is the stimulus for all the changes I am contemplating for the journal which I am narrating in this editorial.

1. Keeping in mind the developments internationally our journal will hence forth use online manuscript submission and electronic peer review system. (www.journalonweb.com). It is going to reproduce the function of an editorial office on the web, providing individualized password protected area of each authority (Author, Reviewer, Editor and Staff), Double-blind peer review, automated email notifications and reminders and MEDLINE links for editors and referees. The system requires web-browser and internet connection only and there no need of any special software or computer resources. With this change, there will be accessibility from any PC. No set business hours - Articles can be received and processed at any hour of the day, faster clerical work, reduced submission - decision time and authors can check and track the submitted articles. With this I am also contemplating best use of referees both near and far and their time, avoiding paper work and global authorships and referencing (Figure 1).

**Figure 1:**
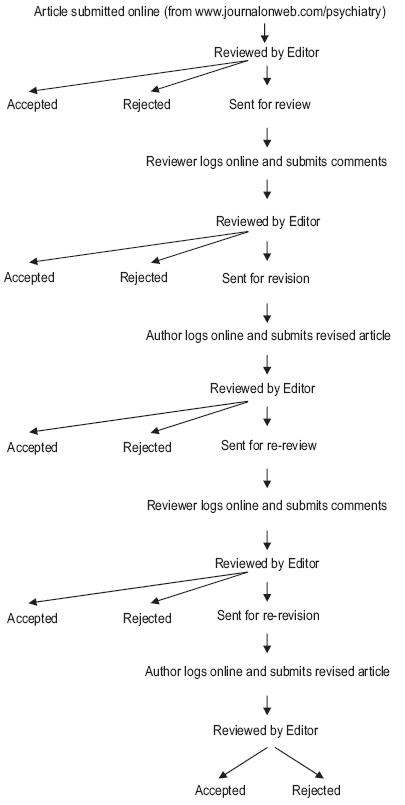
The online review process of Indian Journal of Psychiatry
2. Web site is being designed and should be available to all by early May, 2007. The full text of the articles will be available from www.indianjpsychiatry.org. We are aware that Researchers, as authors, require access to the largest possible audience to disseminate their findings; researchers, as readers, need the broadest possible access to the relevant literature. To the avoid the time lag the articles can be “published” - posted on the Web - as soon as it is accepted and prepared for publication. It is likely to provide faster dissemination of scientific information by expediting peer review, editorial and production work and distribution. We are also aiming at interactivity and reference linking - linking to other electronic information, such as links between reference citations and article abstracts and between references to material on the Web and the remote sites themselves. The availability of articles online via Open Access carry higher impact rates than proprietary only and print articles and we hope to reach the desired goal of NIH indexing of our journal at the earliest.
3. The site being developed will have very innovative features to name a few
  - Facility to search across the full text of multiple journals.
  - Facility to submit comments / remarks on published articles.
  - Ability to go to the other sites via reference and external linking.
  - Linking from other sites so that visitors can reach journal site.
  - International norms in e-publishing: Metatags for articles (e.g., DC metadata), user statistics, site structure (e.g., OpenURL)
4. We have added special section on Literary Psychiatry and Dr. Ajith V. Bhide will be the section editor. I am open for any new innovative idea or section and look forward to your contribution.

I am confident the esteemed IPS membership appreciates the changes being brought about. True to the paradigm ‘Only thing constant is change’, I do hope the new functional changes will stand the test of time. I am grateful to the president, the secretary and the office bearers who have consented to the changes. I am indebted to the help of associate, deputy and assistant editors who have helped the immensely. The editorial board, journal committee, National and International Advisory board, statistical consultants and the esteemed membership of IPS will be my guides. Dr. Sahu of Medknow Publications, Mumbai has been entrusted with an immense task and working with him has been a very pleasant experience for me and confident that this association will prove a boon for the big things all of us are keeping in mind. This is an opportunity for a new beginning and I seek the helping hand of one and all in this pleasant task.

Long Live IPS.

